# Books and Pamphlets Received

**Published:** 1885-07-20

**Authors:** 


### BOOKS AND PAMPHLETS RECEIVED.

*An Epitome of Eye, Ear, Throat, and Nose Diseases, for Student and Practitioner.* Illustrated with wood cuts and photographs. By Arthur G Hobbs, M.D., Professor of Ophthalmology, Otology, and Laryngotomy in the Southern Medical College, Atlanta, Ga. Philadelphia: Samuel M. Miller, M.D., Medical Publisher. 1885.

With the prominent example of Prof. J. J. Chisolm in recommending to the general practitioner a thorough study of this class of diseases, so that he may render the ordinary services to his patients in this department, without the necessity of turning them over to specialists, this undertaking of Professor Hobbs should be hailed by the Medical profession generally as an important addition to our equipment for meeting such exigencies. It has been the Author's aim, as we are informed in his preface, to compress within the narrowest limits such a clear and intelligent account of the diseases in question as is essential for the purpose of practical information ; and while he modestly states that the work is not intended as a text book, nor even as a reference book for the specialists, it is evident that many who have bestowed their attention and study upon these subjects will profit by a careful examination of the contents of this volume. It treats systematically and methodically, in four parts, of the eye, the ear, throat, and nose. The anatomy and physiology of each are given succinctly and clearly at the outset of each division, followed by the mode of examining the organs, and the means by which applications are made to the respective parts, with the treatment proper in the several diseases of the different tissues. A distinct outline is given of what is requisite to be known by those undertaking the treatment of the affections of the eye, ear, throat, and nose ; and any student or practitioner who may become thoroughly acquainted with the details presented under the various headings will go forth well furnished for the work in those several departments.

Few specialists in this field have made such progress in knowledge as not to derive great assistance from the practical instructions contained in this volume, and the simplicity and clearness of the descriptions renders them at the same time comprehensible by those who have not devoted special attention to the diseases of those organs.

We are not aware that any other work has been presented to the Medical public covering the same ground that is occupied by this volume, and it is a subject of congratulation that Professor Hobbs has appreciated the wants of the profession in giving the results of his large experience in the management of these diseases, as well as the teachings of others prominent in this specialty. Not only will his own classes in the SOUTHERN MEDICAL COLLEGE profit by this work, but the students of other Medical Institutions throughout our country will derive great advantage in studying it while attending the regular courses of Lectures in their respective schools.

The recently graduated physician, and, doubtless, many who have been practicing for several years, will also find it a very valuable companion in the treatment of the ordinary affections of these organs, which are encountered in their daily service ; and, after a most careful examination of its contents, we feel warranted in putting it among the most important contributions that have been made to the practical resources of this progressive age.

---

*The International Encyclopœdia of Surgery;* a systematic treatise of the theory and practice of Surgery, by authors of various nations Edited by John Ashurst, jr., M. D , Professor of Clinical Surgery in the University of Pennsylvania. Illustrated with chromo-lithographs and wood cuts ; in six volumes. Vol. V. New York : Wm. Wood & Co. 1884.

This is a large volume, of more than 1,200 pages, being the fifth in a series which is to constitute the great International Encyclopœdia of Surgery, the contents to be furnished by advanced minds from various nations, edited by Dr. Ashurst, of the University of Pennsylvania. "This volume continues the discussion of Surgical affections, arranged according to the regions of the body in which they are observed, embracing articles on injuries, and on diseases of the head ; on those of the eyes, of the ears, and of the nose ; on those of the face, cheeks, and lips ; on those of the mouth, fauces, tongue, palate, and jaws ; on the Surgery of the teeth ; on injuries and diseases of the neck, and of the air-passages ; on injuries of the chest ; on diseases of the breast ; on injuries and diseases of the abdomen, and on hernia. One or more large volumes will be required to complete the ground yet to be covered."

Our space will not admit of such detailed account of the volume before us as its merits demand. It is evident, from a cursory view, that, as in the volumes that have preceded it, the subjects are most ably and thoroughly discussed. The design appears to be a full and complete account of all that is known upon the subjects treated up to date. As a work of reference, it is manifest that the International Encyclopœdia of Surgery will be full and satisfactory, even for the demands of the most thorough investigator, in all departments, and in all that pertains to the theory and practice of Surgery up to the present time. It is certainly a great and extensive work, and must prove immensely useful to, and popular with, Surgeons everywhere.

*A Theoretical and Practical Treatise on the Hemorrhoidal Disease*, giving its History, Nature, Causes, Pathology, Diagnosis, and Treatment. By William Bodenhamer, A M., M.D. Illustrated by two chromo lithographic plates and thirty one wood cuts. New York : Wm. Wood & Co., 58 La-Fayette Place. 1884.

Any work calculated to throw light upon this perplexing class of affections will be welcomed by the profession. Few practitioners, it must be conceded, understand rectal diseases, and so great the antipathy, on the part of most medical men, to treating them, that too frequently they are turned over to specialists, many of whom are really ignorant even of the anatomy of the parts with which they so recklessly deal. The author of the above work claims to have given "a summary account of the theory and the practice of the Ancients as well as that of the Moderns, on this disease, with all the changes and the improvements which have been made, from time to time, down to the present period, together with his own experience of many years. . . It will be found to be a complete encyclopœdia upon the subject of hemorrhoids."

*A Handbook of Pathological Anatomy and Histology*, with an introductory section of Post Mortem Examinations and the Methods of Preserving and Examining Diseased Tissues. By Francis Delafield, M. D., Professor of Pathology and Practical Medicine, College of Physicians and Surgeons, New York ; and T. Mitchell Prudden, M. D., Director of the Physiological and Pathological Laboratory of the Alumni Association of the College of Physicians and Surgeons, New York ; Lecturer on Normal Histology in Yale College. New York : Wm. Wood & Co., 58 LaFayette Place. 1885. Cloth. 575 large octavo pages ; neatly and elegantly printed.

It is a work of great interest, especially to the progressive medical man. It "comprises instruction on the methods of making post mortem examinations, of preserving diseased tissues, and of preparing them for microscopical examination, and of preparing and examining bacteria ; an account of such general processes as inflammation and degeneration ; a description of tumors, of lesions of all the different parts of the body ; of the general diseases ; of violent deaths, and of deaths from poisoning," &c. The work is beautifully illustrated, and evinces great research and marked ability on the part of the authors.

